# Water from air: an overlooked source of moisture in arid and semiarid regions

**DOI:** 10.1038/srep13767

**Published:** 2015-09-08

**Authors:** Theresa A. McHugh, Ember M. Morrissey, Sasha C. Reed, Bruce A. Hungate, Egbert Schwartz

**Affiliations:** 1 U.S. Geological Survey, Southwest Biological Science Center, Moab, UT, USA; 2 Center for Ecosystem Science and Society, Northern Arizona University, Flagstaff, AZ, USA; 3 Department of Biological Sciences, Northern Arizona University, Flagstaff, AZ, USA

## Abstract

Water drives the functioning of Earth’s arid and semiarid lands. Drylands can obtain water from sources other than precipitation, yet little is known about how non-rainfall water inputs influence dryland communities and their activity. In particular, water vapor adsorption – movement of atmospheric water vapor into soil when soil air is drier than the overlying air – likely occurs often in drylands, yet its effects on ecosystem processes are not known. By adding ^18^O-enriched water vapor to the atmosphere of a closed system, we documented the conversion of water vapor to soil liquid water across a temperature range typical of arid ecosystems. This phenomenon rapidly increased soil moisture and stimulated microbial carbon (C) cycling, and the flux of water vapor to soil had a stronger impact than temperature on microbial activity. In a semiarid grassland, we also observed that non-rainfall water inputs stimulated microbial activity and C cycling. Together these data suggest that, during rain-free periods, atmospheric moisture in drylands may significantly contribute to variation in soil water content, thereby influencing ecosystem processes. The simple physical process of adsorption of water vapor to soil particles, forming liquid water, represents an overlooked but potentially important contributor to C cycling in drylands.

In drylands, precipitation is so low relative to demand that the availability of water represents the primary control over biological processes^1^. Accordingly, understanding both the sources of dryland water and how water is accessed is critical for understanding and forecasting arid and semiarid ecosystem function. Drylands cover 41% of Earth’s terrestrial surface^2^ and represent the planet’s largest biome^3^. Thus, improving our understanding of the primary factor controlling dryland function – water availability – is important at the global scale. This is particularly true with regard to the global C cycle as, due to their large spatial extent, dryland soils store nearly twice as much soil organic C as temperate forest soils^4^,^5^. Recent research also suggests that C turnover rates in semiarid ecosystems are an increasingly dominant driver of inter-annual variability in the global C cycle^6^.

This significant role in global biogeochemical cycles and the central influence of water in regulating activity has generated interest in the relationships between precipitation and C cycling in dryland ecosystems^7^,^8^,^9^,^10^. Climate change in the form of altered precipitation timing and amount could alter dryland biota and biogeochemical cycling^11^,^12^. However, dryland ecosystems obtain water from sources other than precipitation and, in contrast to the many studies exploring the links between precipitation and dryland C cycling, almost nothing is known about how soil water derived from atmospheric water vapor influences dryland communities and their activity, nor about how climate change will affect this water input and its interactions with soil C cycling. This knowledge is particularly relevant as increased temperature results in increased capacity of the atmosphere to hold more water^13^,^14^.

In the absence of precipitation, there are three primary mechanisms for the addition of water to surface soils: fog deposition, dew formation, and water vapor adsorption^15^. Fog occurs only when atmospheric water vapor concentrations reach saturation, an uncommon condition in most drylands (but see reference^16^). Dew is formed when the surface temperature is lower than or equal to the dewpoint temperature, at which time water vapor from the air in contact with the cold soil surface condenses to form dew. While dew has been suggested to be an important source of water in some drylands^17^,^18^,^19^, there are many systems and seasons where dew formation is likely to be rare^15^,^20^. In contrast, the formation of liquid water in soil pores resulting from the adsorption of water vapor from air can occur any time the relative humidity in the soil atmosphere is lower than the relative humidity of the overlying air^15^ – in other words, when soil air is drier than the overlying atmosphere. Environmental conditions are such that this phenomenon could take place frequently in many dryland regions throughout the world^15^, constituting an important link in the water cycle of arid and semiarid regions^21^,^22^,^23^. Yet, the limited knowledge we do have about the ecological role of water vapor adsorption – as a liquid water source for vascular plants^22^,^23^ and as a driver of litter decomposition^24^,^25^,^26^ – comes from coastal regions that experience high relative humidity^20^,^27^. Strong water limitation in drylands means that soil water inputs from adsorption of atmospheric water vapor could be an important driver of soil biogeochemistry, yet whether the phenomenon is quantitatively significant and ecologically relevant is not known.

To test for the occurrence of water vapor adsorption and examine the conditions under which it occurs, we conducted two sets of laboratory experiments. First, ^18^O-enriched water vapor was added to the atmosphere of sealed jars containing field dry soil from a semiarid grassland. Temperature and relative humidity were monitored over a three-day period as soils experienced simulated diurnal temperature fluctuations (23 °C for 4 h, 40 °C for 8 h, 23 °C for 4 h, and 10 °C for 8 h) typical for an arid region. Through the addition of ^18^O-enriched water vapor to the atmosphere (0, 50, 100, or 500 μg fully evaporated into ~1 L headspace), we were able to trace the movement of water vapor into the soil. Addition of water vapor to the atmosphere increased the atmospheric relative humidity (Fig. 1a) and soil gravimetric water content (Fig. 1b). The increase in soil gravimetric water content was due to vapor adsorption, as the soil water ^18^O atom percent excess (APE) confirmed the transfer of moisture from the atmosphere to the soil (Fig. 1c). Further, the amount of water vapor adsorbed (calculated from ^18^O APE) was linearly related to the gravimetric moisture content of the soil (r^2^ = 0.91, p < 0.00001). Across all treatments, 35–50% of the water added to the atmosphere was adsorbed. Water vapor adsorption occurred at atmospheric relative humidity values ranging from 20–60% (Fig. 1a,b), which are consistent with humidity values reported for arid and semiarid lands across the globe^28^. Because soil temperatures were well above dewpoint temperatures, the transfer of water from the atmosphere to the soil could not have occurred through dew.

A second laboratory experiment was performed to examine the influence of temperature on water vapor adsorption and the extent to which it regulates soil microbial activity. Field dry soil was incubated for three days under the simulated diurnal temperature fluctuations previously described, or at a constant temperature of either 23 or 40 °C. Water vapor was added to the atmosphere of treatment jars (500 μg fully evaporated into ~1 L headspace), while experimental controls received no water addition. Water vapor adsorption was confirmed by an increase in the gravimetric water content of the soils over the course of the incubation. Relative to their moisture content at the initiation of the experiment (2.3%), the average moisture content of soils in the water vapor adsorption treatment increased by 65.9% for the simulated diurnal treatment, 63.3% for the 23 °C treatment, and 59.9% for the 40 °C temperature regime. Thus, water vapor adsorption occurred under all temperature regimes, and the concomitant increases in CO_2_ efflux (Fig. 2) indicate that vapor adsorption stimulated soil CO_2_ production. The effect of water vapor adsorption was significantly greater in soils experiencing simulated diurnal fluctuations (p < 0.0001, Fig. 2). Overall, temperature explained 23% of the variation in soil CO_2_ efflux rates, while water vapor addition explained 47% (two-way ANOVA). These results suggest that temperature may have opposing effects on microbial activity in dryland soils. On one hand, higher temperatures, which increase molecular diffusion rates, will lead to greater respiration activity but also deplete soils of scarce moisture. On the other hand, colder temperatures will allow more water vapor adsorption, thereby increasing soil moisture and microbial habitat in soil. Maximum respiration rates may be observed when soils are warming up early in the morning, as they still contain moisture trapped through water vapor adsorption, yet are warm enough to support high microbial activity. Others have also observed peaks in respiration during the relatively cool morning hours^29^, and these results, which challenge commonly held beliefs about the relationship between respiration and temperature, could be explained by the movement of water from air to soil. Future climate conditions could alter the importance of water vapor adsorption in dryland function, not only through changes in precipitation and relative humidity, but also via interactions with temperature.

We also explored atmospheric water inputs in the field. At a semiarid grassland in northern Arizona, USA (Supplementary Fig. S1), changes in atmospheric relative humidity, soil moisture, and soil respiration were monitored over a 24-h period during the dry season when native grasses were dormant. We observed a strong diurnal pattern in atmospheric relative humidity that was correlated with temperature, and as expected, during the late night and early morning when temperatures were lowest, relative humidity was highest. There was a strong positive correlation between atmospheric relative humidity and soil moisture (r^2^ = 0.42, p < 0.001). Relative humidity varied from 14 to 49%, gravimetric soil moisture content varied from 0.36 to 4.79%, and both were generally higher during the morning hours. These findings are consistent with other studies showing that diurnal fluctuations in water vapor adsorption by soil during the summer season follow fluctuations in atmospheric relative humidity^22^,^30^,^31^. The increase in the moisture content of the top five centimeters of soil, in conjunction with no associated changes in the moisture content of deeper soil layers, indicates that the water addition was the result of atmospheric inputs and not simply the redistribution of water within the soil profile. Though the resolution of our temperature measurements did not allow us to determine if dew formation also contributed to the observed increase in moisture and activity, the robust relationship between soil moisture and relative humidity – in combination with the lack of moisture input from other sources – provides strong evidence that atmospheric water was added to soil in this grassland ecosystem.

While we focused our study efforts on bare patches of soil within a grassland ecosystem, it is important to consider the microclimate that develops within the plant canopy^32^ and how these conditions impact the addition of non-rainfall water. In a semiarid grassland in southeastern Spain, net water gains from water vapor adsorption were found to be higher in bare soil than in soil under *Stipa tenacissima* tussocks, as vegetation limits fluctuations in atmospheric relative humidity near the soil^30^.

In order to test the hypothesis that increased soil moisture from non-rainfall water inputs was the main driver of soil CO_2_ efflux in the field (Supplementary Fig. S2), we conducted a path analysis to determine how soil temperature and moisture affected soil respiration. A theory-constrained full model was developed to represent all hypothetical relationships between soil temperature, soil moisture, and soil CO_2_ efflux within our system. Using the experimental data, a reduced model was attained by sequentially removing paths in order of largest probability value until all remaining paths were significant (Fig. 3). These results are consistent with a scenario in which low temperatures enable the movement of water vapor from air to soil, and the subsequent increase in soil moisture, in turn, stimulates soil CO_2_ efflux rates. This demonstration of increased CO_2_ efflux in the laboratory and under field conditions provides strong evidence that atmospheric water influences the activity of soil microorganisms.

While we know little about the biological significance of atmospheric water vapor on soil microbial activity, it has been well documented that very small moisture inputs can activate ecosystem processes in dryland regions^33^,^34^. Indeed, small inputs of water can drive dryland community composition and biogeochemical cycles^8^,^10^,^12^. Thus, liquid water from the atmosphere has the potential to be a critical source of water for dryland organisms. More regions worldwide are expected to experience reduced rainfall as a result of climate change^35^, a scenario where non-rainfall atmospheric water inputs could become an increasingly important source of soil moisture as higher amounts of atmospheric water vapor are predicted globally^13^,^14^. Although the nature of the interactions among temperature, atmospheric humidity, and soil humidity require further inquiry, our data suggest that these unexplored changes could impact dryland C fluxes. In particular, our laboratory and field results demonstrated that non-rainfall addition of moisture to soil can occur across a large range of relative humidity values, and established that this phenomenon influences soil C cycling in dryland soils. Future research should aim to address the applicability of these observations to global drylands, quantify the role of dew versus water vapor adsorption inputs, and determine the contribution of this water source to daily and seasonal C fluxes.

## Methods

### Laboratory Experiments

In the first laboratory experiment, 30 g samples of sieved (2 mm) soil from the grassland were placed in 1000 ml sealed Nalgene jars also containing a hygro-thermometer (Extech Instruments; Nashua, NH) and a small reservoir with either 0, 50, 100, or 500 μg of ^18^O-enriched water (2 atom%). Triplicate jars for each treatment were then subjected to temperature cycling designed to simulate diurnal temperature fluctuations typical of an arid environment: 4 h at 23 °C, 8 h at 40 °C, 4 h at 23 °C, 8 h at 10 °C, repeated over a 72-h period. Immediately prior to each temperature transition, relative humidity and temperature values were recorded. Gravimetric moisture content was determined for each soil sample at the end of the experiment (105 °C for 24 h). Water was extracted from soil samples using a cryogenic vacuum line^36^. Extracted samples were then analyzed for δ^18^O using a G1102-i isotopic H_2_O cavity ring down spectrometer (Picarro Inc; Santa Clara, CA). The amount of water vapor adsorbed (H_2_O_WVA_) was calculated using the known isotope compositions of the water added to the atmosphere (2 atom% ^18^O), the native soil water (~natural abundance: 0.19 atom% ^18^O), and the isotopic mass balance equation where:


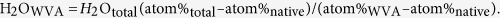


For the second laboratory experiment, 30 g samples of sieved (2 mm) soil from the grassland were placed in each of 30 sealed Mason jars (946 ml). Half of the jars included a small reservoir with 500 μg of water, while the other half experienced no atmospheric water addition. The jars were then equally divided so that five replicate jars were incubated for 72 h at high (40 °C) or moderate (23 °C) temperature, or the temperature cycling previously described. Gas samples (5 ml) were collected from the headspace of each jar 0, 52, and 72 h after the initiation of the experiment using an air-tight syringe inserted through a septum on the lid of each jar. Headspace samples were analyzed for CO_2_ concentration using an infrared gas analyzer model L1-6262 (LI-COR Biosciences; Lincoln, NE). All samples showed a linear increase in CO_2_ concentration over time, and production rates (μg CO_2_-C g soil^−1^ day^−1^) were calculated using linear regression (median correlation coefficient = 0.92).

### Site Description and Field Study

The field site is a semiarid, high-desert grassland near Flagstaff, AZ, USA (35°34′20′N, 111°34′4′W, 1755 m above sea level, 230 mm of rain annually). The dominant plants are perennial grasses (*Bouteloua eriopoda*, *Bouteloua gracilis*, *Sporobulus cryptandrus*, and *Pleuraphis jamesii*) with few shrubs (*Ericameria nauseousa*, *Gutierrezia sarothrae*). Soils are cindery and are classified in the U.S. Department of Agriculture Soil Taxonomic Subgroup of Typic Haplustolls. There is a Campbell Scientific (Logan, UT) weather station located at this field site that collects temperature, moisture, and humidity data in addition to other environmental variables.

In early July of 2013, prior to the arrival of monsoon precipitation and during a period when grasses were physiologically dormant, a 24-hour observational study was conducted in order to characterize diurnal soil moisture fluctuations and CO_2_ efflux rates. Soil respiration measurements were taken with a LI-8100A Automated Soil Flux System (LI-COR Biosciences; Lincoln, NE). Ten soil collars (50 cm diameter) were installed in plant interspaces throughout the site, and CO_2_ efflux measurements were taken at these locations every hour for 24 h. One soil core (5 cm diameter and depth) was collected every hour during the 24-h period for gravimetric moisture determination. Soil samples were sieved (2 mm) and weighed in the field, then carefully transported back to the laboratory where they were dried (105 °C for 24 h) and reweighed.

### Data Analysis

Treatment effects in the laboratory experiments were assessed with one- or two-way ANOVA in JMP Pro 9.0.2 (Cary, NC). Linear regressions were performed in R (http://www.R-project.org). Path analysis was performed with SPSS Amos 22, and the path model was a good fit to the data (Chi square = 0.635, p = 0.426).

## Additional Information

**How to cite this article**: McHugh, T. A. *et al.* Water from air: an overlooked source of moisture in arid and semiarid regions. *Sci. Rep.* **5**, 13767; doi: 10.1038/srep13767 (2015).

## Supplementary Material

Supplementary Figures S1 and S2

## Figures and Tables

**Figure 1:**
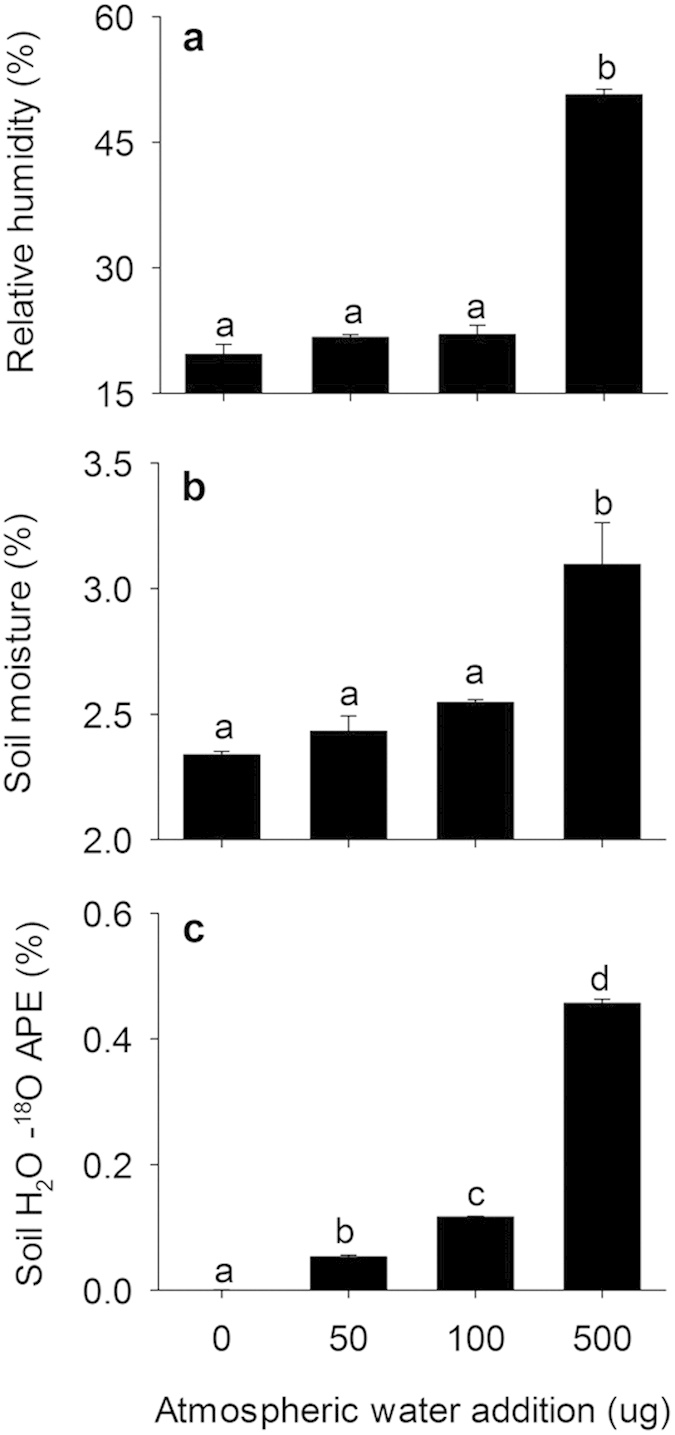
Atmospheric relative humidity (**a**) soil gravimetric moisture content (**b**) and soil water ^18^O atom percent excess (**c**) in response to atmospheric water vapor addition. Error bars are standard error for means (n = 3). Significant differences as determined by one-way ANOVA and Tukey’s post-hoc test are indicated using lowercase letters (α = 0.05).

**Figure 2:**
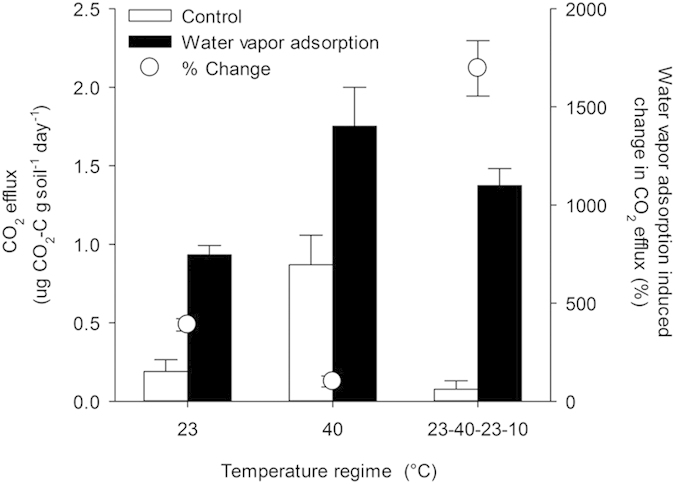
Soil CO_2_ efflux rates across temperature regimes for control and water vapor adsorption treatment. Error bars are standard error for means (n = 5). The effect of water vapor adsorption (percent change relative to control) is shown with white circles.

**Figure 3:**
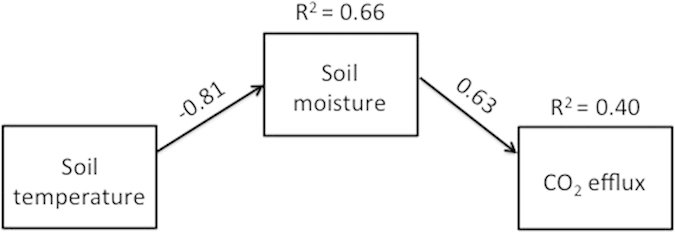
Path diagram displaying the role of soil temperature and soil moisture in regulating soil CO_2_ efflux rates under a reduced model. Arrows represent unidirectional causal relationships. The amount of variation that can be explained by the model is indicated by the R^2^ values associated with each response variable. Standardized path coefficients (r) associated with each arrow reflect the strength of each relationship (p  <  0.001 in both cases). The full model also included the direct assessment of temperature effects on CO_2_ efflux rates, but since that relationship was not statistically significant, no arrow is shown.
