# Supplementary material for: Water from air: an overlooked source of moisture in arid and semiarid regions
Source: Sci Rep. 2015 Sep 8;5:13767. doi: 10.1038/srep13767 (PMC4561883; doi:10.1038/srep13767)
Supplement: Supplementary Figures S1 and S2 [file srep13767-s1.pdf]

Water from air: an overlooked source of moisture in arid and semiarid regions

Theresa A. McHugh, Ember M. Morrissey, Sasha C. Reed, Bruce A. Hungate, Egbert Schwartz

a

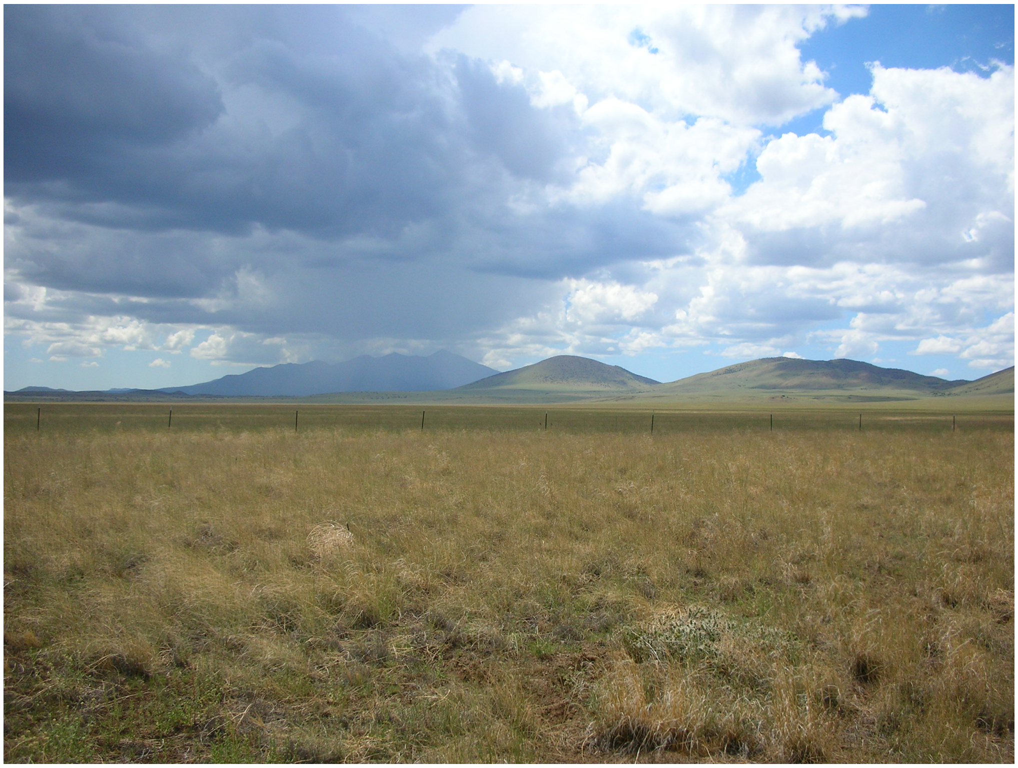

b

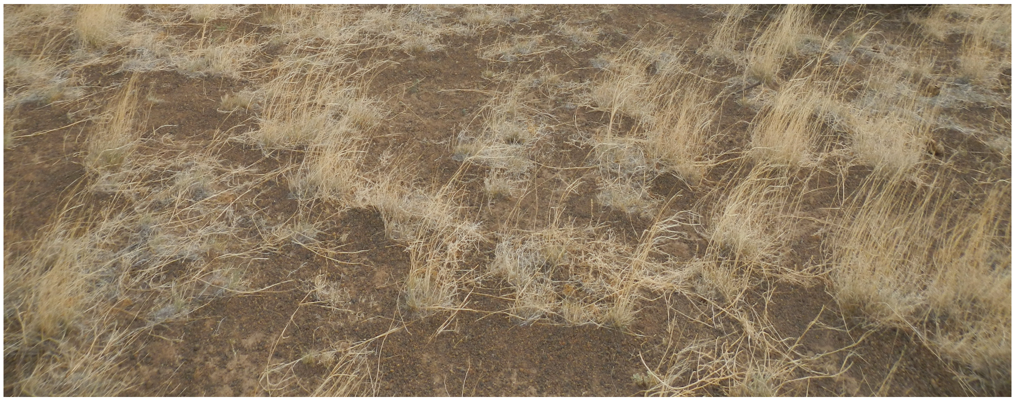

Supplementary Figure S1 | Semiarid, high elevation grassland where the field study was conducted (a), and close-up of the soil surface during the study period when plants were dormant (b).

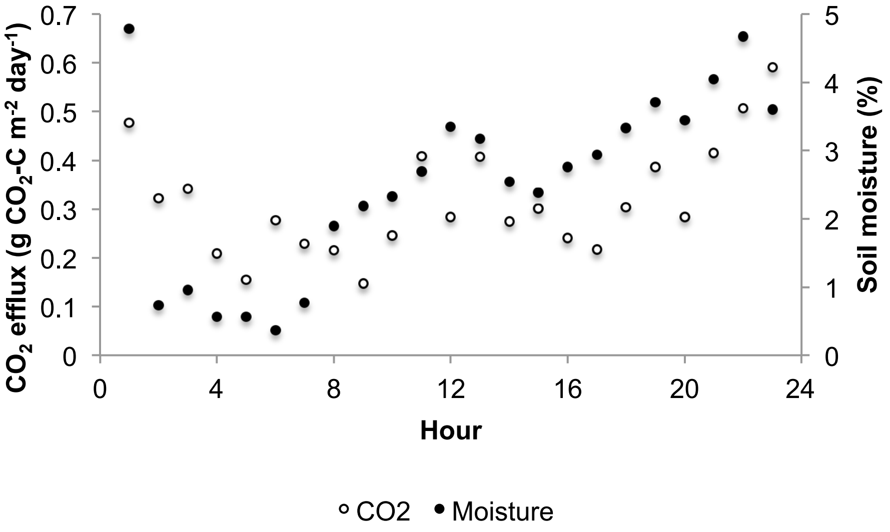

Supplementary Figure S2 | Mean CO_2 efflux rates and soil moisture contents in the field during the 24-hour study.
